# Supplementary material for: State-wide random seroprevalence survey of SARS-CoV-2 past infection in a southern US State, 2020
Source: PLoS One. 2022 Apr 27;17(4):e0267322. doi: 10.1371/journal.pone.0267322 (PMC9045671; doi:10.1371/journal.pone.0267322)
Supplement: S1 Table — (DOCX) [file pone.0267322.s001.docx]

**S1 Table. Comparison of characteristics of participants and non-participants, in a random sample of adults, Arkansas, May – December 2020.**

| Characteristics | Participants (%) | non-Participants (%) | **P*-value |
| --- | --- | --- | --- |
| Age (yrs.) |  |  |  |
| 18-49 | 883 (52.2) | 754 (57.8) | <0.01 |
| 50+ | 809 (47.8) | 551 (42.2) |  |
| Sex |  |  |  |
| Female | 1077 (63.6) | 744 (57.1) | <0.001 |
| Male | 617 (36.4) | 557 (42.7) |  |
| Race/Ethnicity |  |  |  |
| Non-Hispanic Whites | 1261 (80.2) | 845 (75.4) | Referent |
| Non-Hispanic Blacks | 197 (12.5) | 197 (17.6) | <0.005 |
| Hispanics | 91 (5.8) | 56 (5.0) | <0.001 |
| Other | 24 (1.5) | 22 (2.0) | <0.001 |

*Margin = 0.1 using the Farrington-Manning score method. A small P-value suggest the null hypothesis, stating the groups differ by more than the tolerably small amount or margin of 10%, should be rejected.
