# Supplementary material for: State-wide random seroprevalence survey of SARS-CoV-2 past infection in a southern US State, 2020
Source: PLoS One. 2022 Apr 27;17(4):e0267322. doi: 10.1371/journal.pone.0267322 (PMC9045671; doi:10.1371/journal.pone.0267322)
Supplement: S2 Table — The analytic dataset of the 2020 ARCASS and data dictionary is available in the following doi: Cardenas, Victor (2022): public.csv. figshare. Dataset and dictionary. https://doi.org/10.6084/m9.figshare.19119524.v1. (DOCX) [file pone.0267322.s002.docx]

Supplemental Table 2 is hosted on figshare: <https://figshare.com/articles/dataset/public_csv/19119524/1>
