# Supplementary material for: State-wide random seroprevalence survey of SARS-CoV-2 past infection in a southern US State, 2020
Source: PLoS One. 2022 Apr 27;17(4):e0267322. doi: 10.1371/journal.pone.0267322 (PMC9045671; doi:10.1371/journal.pone.0267322)
Supplement: Attachment — Submitted filename: Reviewer.pdf [file pone.0267322.s005.pdf]

Reviewer

This study focused on estimating the number of Arkansans residents infected with SARS-CoV-2 between May and December 2020 and also to assess the determinants of infection. This was carried out by surveying the seroprevalence of, a statewide population-based random-digit dial sample of non-institutionalized adults in Arkansas. The outcome was past Covid-19 infection measured by serum antibody test . Notably, the seropositivity was significantly elevated among non-Hispanic black , Hispanic and

The method on how All sera were tested for IgG antibodies that target receptor binding domain of the spike protein of the SARS CoV-2 using the Beckman Coulter DxI instrument should be explained.

Major issues:

Overall, the data is promising, but the novelty of this study is relatively weak because the outcomes of the results obtained is not clearly explained.

Minor issues:

1. The introduction section needs to be worked on and be improved for example

Of these, only seven used random sampling procedures so that every person in the target population had “a known, non-zero probability of being included in the sample

A comma interferes with the flow.

The data obtained in this work are of interest for infectious disease specialists. The research was carried out using adequate methods and the manuscript may be published.
