# Supplementary material for: State-wide random seroprevalence survey of SARS-CoV-2 past infection in a southern US State, 2020
Source: PLoS One. 2022 Apr 27;17(4):e0267322. doi: 10.1371/journal.pone.0267322 (PMC9045671; doi:10.1371/journal.pone.0267322)
Supplement: Attachment — Submitted filename: PONE-D-21-30543 (1)_Plos One.pdf [file pone.0267322.s006.pdf]

PLOS ONE

State-wide random seroprevalence survey of SARS-CoV-2 past infection in a southern US State, 2020

--Manuscript Draft--

**Manuscript Number:**	PONE-D-21-30543
**Article Type:**	Research Article
**Full Title:**	State-wide random seroprevalence survey of SARS-CoV-2 past infection in a southern US State, 2020
**Short Title:**	Prevalence of SARS-CoV-2 infection in a Southern US State in 2020
**Corresponding Author:**	Victor M Cardenas, MD, MPH, Cardenas, MD, MPH, PhD, FACE<br>UAMS College of Public Health<br>Little Rock, Arkansas UNITED STATES
**Keywords:**	coronavirus; COVID-19; SARS-CoV-2; cross-sectional studies; serologic surveys; United States
**Abstract:**	The purpose of this cross-sectional study was to estimate the proportion of Arkansas residents who were infected with the SARS-CoV-2 virus between May and December 2020 and to assess the determinants of infection. To estimate seroprevalence, a state-wide population-based random-digit dial sample of non-institutionalized adults in Arkansas was surveyed. Exposures were age, sex, race/ethnicity, education, occupation, contact with infected persons, comorbidities, height, and weight. The outcome was past COVID-19 infection measured by serum antibody test. We found a prevalence of 15.1% (95% CI: 11.1%, 20.2%) by December 2020. Seropositivity was significantly elevated among participants who were non-Hispanic Black, Hispanic (prevalence ratio [PRs]:1.4 [95% CI: 0.8 , 2.4] and 2.3 [95% CI: 1.3, 4.0], respectively), worked in high-demand essential services (PR: 2.5 [95% CI: 1.5, 4.1]), did not have a college degree (PR: 1.6 [95% CI: 1.0, 2.4]), had an infected household or extra-household contact (PRs: 4.7 [95% CI: 2.1, 10.1] and 2.6 [95% CI: 1.2, 5.7], respectively), and were contacted in November or December (PR: 3.6 [95% CI: 1.9, 6.9]). Our results indicate that by December 2020, one out six persons in Arkansas had a past SARS-CoV-2 infection.
**Order of Authors:**	Victor Manuel Cardenas, MD, MPH, PhD
	Joshua Lauren Kennedy, MD
	Mark L Williams, PhD
	Wendy N Nembhard, MPH, PhD
	Namvar Zohoori, MD, MPH, PhD
	Ruofei Du, PhD
	Jing Jin, MPH
	Danielle B. Boothe, MPH
	Lori Ann Fischbach, MPH, PhD
	Catherine Kirkpatrick, BS
	Zeel Nilesh Modi, BA
	Katherine Caid, BS
	Shana Owens, PhD
	J Craig Forrest, PhD
	Laura P James, MD
	Karl W Boehme, PhD
	Ericka Olgaard, DO

Powered by Editorial Manager® and ProduXion Manager® from Aries Systems Corporation

	Stephanie F Gardner, PhD, Ed.D.
	Benjamin C Amick, PhD
**Additional Information:**	
**Question**	**Response**
**Financial Disclosure**<br><br>Enter a financial disclosure statement that describes the sources of funding for the work included in this submission. Review the submission guidelines for detailed requirements. View published research articles from *PLOS ONE* for specific examples.<br><br>This statement is required for submission and **will appear in the published article** if the submission is accepted. Please make sure it is accurate.<br><br>**Unfunded studies**<br>Enter: *The author(s) received no specific funding for this work.*<br><br>**Funded studies**<br>Enter a statement with the following details:<br>• Initials of the authors who received each award<br>• Grant numbers awarded to each author<br>• The full name of each funder<br>• URL of each funder website<br>• Did the sponsors or funders play any role in the study design, data collection and analysis, decision to publish, or preparation of the manuscript?<br>• **NO** - Include this sentence at the end of your statement: *The funders had no role in study design, data collection and analysis, decision to publish, or preparation of the manuscript.*<br>• **YES** - Specify the role(s) played.<br><br>* typeset	The work was supported through a research contract agreement with the Arkansas Department of Health with funding from the 2020 Coronavirus Relief Fund - CARES Act (VMC, LAF and LJ -PIs of record) and by grant UL1 TR003107 from the National Center for Advancing Translational Sciences (NCATS) (LJ -PI).
**Competing Interests**<br><br>Use the instructions below to enter a competing interest statement for this submission. On behalf of all authors, disclose any competing interests that could be perceived to bias this work—acknowledging all financial support and any other relevant financial or non-	The authors have declared that no competing interests exist.

financial competing interests.<br><br>This statement is required for submission and **will appear in the published article** if the submission is accepted. Please make sure it is accurate and that any funding sources listed in your Funding Information later in the submission form are also declared in your Financial Disclosure statement.<br><br>View published research articles from *PLOS ONE* for specific examples.<br><br>**NO authors have competing interests**<br><br>Enter: *The authors have declared that no competing interests exist.*<br><br>**Authors with competing interests**<br><br>Enter competing interest details beginning with this statement:<br><br>*I have read the journal's policy and the authors of this manuscript have the following competing interests: [insert competing interests here]*<br><br>* typeset	
**Ethics Statement**<br><br>Enter an ethics statement for this submission. This statement is required if the study involved:<br><br>• Human participants<br>• Human specimens or tissue<br>• Vertebrate animals or cephalopods<br>• Vertebrate embryos or tissues<br>• Field research<br><br>Write "N/A" if the submission does not require an ethics statement.<br><br>General guidance is provided below.<br>Consult the submission guidelines for	The protocol was reviewed and approved by the UAMS Institutional Review Board (Protocol 261232).

detailed instructions. **Make sure that all information entered here is included in the Methods section of the manuscript.**<br><br>**Format for specific study types**<br><br>**Human Subject Research (involving human participants and/or tissue)**<br>• Give the name of the institutional review board or ethics committee that approved the study<br>• Include the approval number and/or a statement indicating approval of this research<br>• Indicate the form of consent obtained (written/oral) or the reason that consent was not obtained (e.g. the data were analyzed anonymously)<br><br>**Animal Research (involving vertebrate animals, embryos or tissues)**<br>• Provide the name of the Institutional Animal Care and Use Committee (IACUC) or other relevant ethics board that reviewed the study protocol, and indicate whether they approved this research or granted a formal waiver of ethical approval<br>• Include an approval number if one was obtained<br>• If the study involved *non-human primates*, add *additional details* about animal welfare and steps taken to ameliorate suffering<br>• If anesthesia, euthanasia, or any kind of animal sacrifice is part of the study, include briefly which substances and/or methods were applied<br><br>**Field Research**<br><br>Include the following details if this study involves the collection of plant, animal, or other materials from a natural setting:<br>• Field permit number<br>• Name of the institution or relevant body that granted permission	
**Data Availability**<br><br>Authors are required to make all data underlying the findings described fully available, without restriction, and from the time of publication. PLOS allows rare	No - some restrictions will apply

exceptions to address legal and ethical concerns. See the [PLOS Data Policy] and [FAQ] for detailed information.<br><br>A Data Availability Statement describing where the data can be found is required at submission. Your answers to this question constitute the Data Availability Statement and **will be published in the article**, if accepted.<br><br>**Important:** Stating 'data available on request from the author' is not sufficient. If your data are only available upon request, select 'No' for the first question and explain your exceptional situation in the text box.<br><br>Do the authors confirm that all data underlying the findings described in their manuscript are fully available without restriction?	
**Describe where the data may be found in full sentences. If you are copying our sample text, replace any instances of XXX with the appropriate details.**<br><br>• If the data are **held or will be held in a public repository**, include URLs, accession numbers or DOIs. If this information will only be available after acceptance, indicate this by ticking the box below. For example: *All XXX files are available from the XXX database (accession number(s) XXX, XXX.*).<br>• If the data are all contained **within the manuscript and/or Supporting Information files**, enter the following: *All relevant data are within the manuscript and its Supporting Information files.*<br>• If neither of these applies but you are able to provide **details of access elsewhere**, with or without limitations, please do so. For example:<br><br>*Data cannot be shared publicly because of [XXX]. Data are available from the XXX Institutional Data Access / Ethics Committee (contact via XXX) for researchers who meet the criteria for*	All analytic de-identified files are available from the ARCASS database (accession number(s) TBD).

access to confidential data.<br><br>*The data underlying the results presented in the study are available from (include the name of the third party and contact information or URL).*<br>• This text is appropriate if the data are owned by a third party and authors do not have permission to share the data.<br><br>* typeset	
Additional data availability information:	Tick here if the URLs/accession numbers/DOIs will be available only after acceptance of the manuscript for publication so that we can ensure their inclusion before publication.

Powered by Editorial Manager® and ProduXion Manager® from Aries Systems Corporation

Manuscript
Click here to access/download;Manuscript;Manuscriptfile 9_21_21.docx

State-wide random seroprevalence survey of SARS-CoV-2 past infection in a southern US State, 2020

Victor M. Cardenas, MD, MPH, PhD, FACE[1*¶], Joshua L. Kennedy, MD [2, 3, 4], Mark Williams, PhD[5], Wendy N. Nembhard, MPH, PhD, FACE[1], Namvar Zohoori, MD, MPH, PhD[1,6] , Ruofei Du, PhD[7], Jing Jin, MPH[1,7], Danielle Boothe, MPH[1], Lori A. Fischbach, MPH, PhD[1,8], Catherine Kirkpatrick, BS[4], Zeel Modi, BA[4], Katherine Caid, BS[4], Shana Owens, PhD[4], J. Craig Forrest, PhD[9], Laura James, MD[2], Karl W. Boehme, PhD[9,10], Ericka Olgaard, DO[11], Stephanie F. Gardner, PharmD, EdD[12], Benjamin C. Amick III, PhD[1,13] on behalf of the Arkansas Coronavirus Antibodies Seroprevalence Survey team

Affiliations

1 Department of Epidemiology, Fay W. Boozman College of Public Health, University of Arkansas for Medical Sciences, Little Rock, Arkansas

2 Department of Paediatrics, College of Medicine, University of Arkansas for Medical Sciences, Little Rock, Arkansas

3 Department of Internal Medicine, College of Medicine, University of Arkansas for Medical Sciences, Little Rock, Arkansas

4 Arkansas Children's Research Institute, Little Rock, Arkansas

5 Department of Health Behaviour and Health Education, Fay W. Boozman College of Public Health, University of Arkansas for Medical Sciences, Little Rock, Arkansas

6 Arkansas Department of Health, Little Rock, Arkansas

7 Department of Biostatistics, College of Public Health, University of Arkansas for Medical Sciences

8 Los Angeles County Department of Public Health, Outbreak Management Branch, Los Angeles, California

9 Department of Microbiology & Immunology, College of Medicine, University of Arkansas for Medical Sciences, Little Rock, Arkansas

10 Centre for Microbial Pathogenesis and Host Inflammatory Responses, University of Arkansas for Medical Sciences, Little Rock, Arkansas

11 Department of Pathology, College of Medicine, University of Arkansas for Medical Sciences, Little Rock, Arkansas

12 College of Pharmacy, University of Arkansas for Medical Sciences, Little Rock, Arkansas

13 Winthrop P. Rockefeller Cancer Institute, University of Arkansas for Medical Sciences, Little Rock, Arkansas

*Corresponding author

Email vmcardenas@uams.edu (VMC)

¶VMC and JLK are both first co-authors.

¶¶ A list of other members of the Arkansas Coronavirus Antibodies Seroprevalence Survey team is provided in Acknowledgments

Abstract

The purpose of this cross-sectional study was to estimate the proportion of Arkansas residents who were infected with the SARS-CoV-2 virus between May and December 2020 and to assess the determinants of infection. To estimate seroprevalence, a state-wide population-based random-digit dial sample of non-institutionalized adults in Arkansas was surveyed. Exposures were age, sex, race/ethnicity, education, occupation, contact with infected persons, comorbidities, height, and weight. The outcome was past COVID-19 infection measured by serum antibody test. We found a prevalence of 15.1% (95% CI: 11.1%, 20.2%) by December 2020. Seropositivity was significantly elevated among participants who were non-Hispanic Black, Hispanic (prevalence ratio [PRs]:1.4 [95% CI: 0.8 , 2.4] and 2.3 [95% CI: 1.3, 4.0], respectively), worked in high-demand essential services (PR: 2.5 [95% CI: 1.5, 4.1]), did not have a college degree (PR: 1.6 [95% CI: 1.0, 2.4]), had an infected household or extra-household contact (PRs: 4.7 [95% CI: 2.1, 10.1] and 2.6 [95% CI: 1.2, 5.7], respectively), and were contacted in November or December (PR: 3.6 [95% CI: 1.9, 6.9]). Our results indicate that by December 2020, one out six persons in Arkansas had a past SARS-CoV-2 infection.

Introduction

Serologic surveys assess the extent of viral infection at the population-level and can inform the decision-making process for returning to normal activities (1). In the United States (US), most seroprevalence surveys of the SARS-Cov-2 virus, the etiologic agent of COVID-19, were conducted before October 2020 and in non-probability samples (2-18). Of these, only seven used random sampling procedures so that every person in the target population had “a known, non-zero probability of being included in the sample” (19). Only two of the seven studies (6,7) were state-wide.

In this study, we expand on the small set of state-wide seroprevalence studies reporting results of a random sample serologic survey conducted in Arkansas, US. Arkansas has been among the southern states most affected by the fourth wave of the COVID-19 pandemic in the summer of 2021. The study was conducted to 1) provide population-based estimates of COVID-19 prevalence in Arkansas between May and December 2020, and 2) examine the association of age, gender, race/ethnicity, rural residence, contact with suspected infected persons, education, and occupation with past infection with SARS-CoV-2 as measured by IgG antibodies.

Materials and Methods

Study design, study population and data collection

The Arkansas Coronavirus Antibodies Seroprevalence Survey (Arkansas CASS) data were collected as part of a larger survey conducted between May and December 2020. Our study is a cross-sectional study also referred to as a prevalence study (20). The target population was the non-institutionalized adult population of the state. A random sample of the target was obtained as follows. Potential participants were

contacted using random digit dialing of mixed land line and targeted cell phone numbers in Arkansas. Land lines were a random sample of all known land lines in the state. Cell phone numbers were a random sample of active numbers used in Arkansas. Usage in the state was determined by call volume and location where a particular cell phone was used most. The mixed sample of land and cell phone numbers was purchased from a national company (Dynata Inc.) having access to these data and experience doing telephone polling in Arkansas. Samples of phone numbers were received from the company every two weeks.

To collect data, trained research assistants (RAs) called numbers from a list. If an eligible person answered the call, the RA explained that he/she was calling from a health science center and asked if the respondent was interested in answering questions about the COVID-19 pandemic. If the person refused to participate, the RA thanked him/her and proceeded to the next number. If the person reached was only Spanish speaking, an RA fluent in Spanish spoke with the respondent.

After expressing willingness to participate, the RA asked if the respondent was: 18 years or older, a resident of Arkansas, able to understand and speak English or Spanish. The willingness to go forward with the poll was used as implicit consent.

When a participant completed the poll, s/he was asked if s/he would be willing to participate in pandemic research. Those agreeing were informed about the study and, if they wished to continue, were scheduled for a blood draw and an interview. To collect a blood specimen, participants were given the choice of having a trained phlebotomist travel to his/her home (option chosen by 63.7%) or the participant could drive to a nearby local clinic (option chosen by 36.3%).

Participants were first provided the opportunity to consent then complete an interview and then the blood draw. All participants provided e-consent via the Research Electronic Data Capture system (REDCap v 11, Vanderbilt U, Nashville, TN) on a tablet. Interview responses were recorded using REDCap. The questionnaire collected data on age, gender, body weight, height, race/ethnicity, education, occupation, history of COVID-19-like illness, comorbidities, and contacts with persons who might have been infected with COVID-19.

Following interview completion, a 5 mL venous blood specimen was obtained. After completing the blood draw, participants received a $40 gift card. Specimens were collected in labelled clot activated sterile tubes, centrifuged, cold packed, and then shipped the same day to a dedicated central study laboratory using a courier service.

Measure of SARS-CoV-2 infection

The outcome variable was evidence of COVID-19 infection as measured by a positive clinical laboratory test. All sera were tested for IgG antibodies that target receptor binding domain of the spike protein of the SARS CoV-2 using the Beckman Coulter DxI instrument (Brea, CA; Access SARS-CoV-2 IgG chemiluminescence immunoassay) in a CLIA certified clinical laboratory. The Access SARS-CoV-2 IgG immunoassay has a sensitivity (Se) of 93.8% and specificity (Sp) of 100.0% (21).

Protection of human subjects

The protocol was reviewed and approved by the UAMS Institutional Review Board (Protocol 261232).

Data analysis

Potential selection bias was assessed comparing the proportions reporting that someone in their household may have had COVID-19 among those who declined to take part and those participating in the Arkansas CASS. We tested for group equivalence, within a margin of 2.5%, a difference that would be considered significant (22). We used a raking procedure (23) in R (R Core Team, 2017) to obtain post-stratification weights, and computed final weights factoring the probabilities of selection based on age, sex, and race/ethnicity distribution of the state population (24).

We determined that a total sample a size of 1,500 subjects would be required to detect increases of at least twice a baseline prevalence level of 3% with a statistical power of >80%. For statistical analyses, we used the subpopulation of records with complete information on immunoassay, age, gender, and race/ethnicity (n=1,565). We used Taylor series linearization estimators available in SUDAAN version 11 (RTI, Research Triangle Park: NC). We followed the ultimate cluster variance approach assuming sampling with replacement as described elsewhere (25). The reciprocal of a respondent's probability of selection or base weight was multiplied by the post-stratification raking weights to obtain the final sampling weights.

We estimated: 1) an 8-month point prevalence as the proportion of individuals with a past COVID-19 infection during the entire study period (i.e., [$\hat{P}_{t_{(May,Dec)}} = \frac{C_{t_{(May,Dec)}}}{N_{t_{(May,Dec)}}}$], and 2) a two month point prevalence ($\hat{P}_{t_i}$) of COVID-19 infection as the proportion of infected among those specimens collected in November and December (26). Observations were grouped by approximate month of collection into three groups:

May-August, September-October and November-December. Because of the potential for misclassification of the outcome due to imperfect sensitivity, the prevalence of COVID-19 was adjusted following recent recommendations (27).

The exposure variables were age (two categories 18-39, 40+ years), sex (male/female), race/ethnicity (non-Hispanic Whites [NHW], non-Hispanic Blacks [NHB], Hispanics, other), collection period (May-August, September-October, November-December), education (no college/college), rural/urban (28), contact with potential SARS-CoV-2 infected persons, number of persons in the household, and Standard Occupation Codes (29). Occupations were grouped by title according to "essential" service, other occupations, and not working (18).

To obtain estimates of the association of SARS-CoV-2 infection, we estimated the prevalence ratios (PR) and 95% confidence intervals (30). Unadjusted PRs were estimated for potential confounders, and stratified analyses assessed confounding and effect modification. Trends were assessed using the Cochran-Mantel-Haenszel test (31). Adjusted prevalence ratios were estimated using predicted marginals from logistic regression (30). All exposure variables were entered into multivariable models, but only those that meaningfully changed the crude estimates of other exposure variables and were significant at $P \leq 0.05$ were included in the final model. Ordinal variables were treated as pseudo-continuous in the logistic regression models. The appropriateness of the multivariable logistic regression model was assessed using a Wald F Hosmer-Lemeshow goodness-of-fit test (32). All analyses were conducted using SAS (v.9, Cary, NC) and SAS-callable SUDAAN (v. 11, RTI, NC).

The reporting of this study conforms to the STROBE statement (33).

Results

Comparison of respondents and non-respondents

There was no difference among participants and non-participants in the study regarding the proportion that knew or thought a member of the household was infected with SARS-CoV-2 (7.2% (95% CI: 6.7%, 7.9%) and 7.6% (95% CI: 6.4%, 9.0%), respectively. The proportion of all potentially eligible participants taking part in the study was 56.3% (n=1,696), and 1,565 were in the eligible population as described above. Differences between participants and non-participants were within ten percentage points for age, sex and race/ethnicity (supplemental Table).

Supplemental Table: Comparison of characteristics of participants and non-participants, in a random sample of adults, Arkansas, May – December 2020.

Characteristics	Participants (%)	non-Participants (%)	**P*-value
Age (yrs.)			
18-49	883 (52.2)	754 (57.8)	<0.01
50+	809 (47.8)	551 (42.2)	
Sex			
Women	1077 (63.6)	744 (57.1)	<0.001
Men	617 (36.4)	557 (42.7)	
Race/Ethnicity			
Non-Hispanic Whites	1261 (80.2)	845 (75.4)	Referent
Non-Hispanic Blacks	197 (12.5)	197 (17.6)	<0.005
Hispanics	91 (5.8)	56 (5.0)	<0.001
Other	24 (1.5)	22 (2.0)	<0.001

*Margin = 0.1 using the Farrington-Manning score method. A small P-value suggest the null hypothesis, stating the groups differ by more than the tolerably small amount or margin of 10%, should be rejected.

SARS-CoV-2 infection

During the 8-month data collection period, the overall prevalence of past COVID-19 infection was 7.1% (95% CI: 5.8%, 8.7%). The crude prevalence increased

by a factor of 4.2 over time from 3.3% (95% CI: 1.9%, 5.9%) at the end of August to 14.2% (95% CI: 10.4%, 19.0%) at the end of December (Cochran-Mantel-Haenszel test for trend *P*-value <0.0001) (Table 1). Estimates of point prevalence by approximate month of collection are shown in Figure 1.

Table 1: Weighted period prevalence of SARS-CoV-2 past infection and prevalence ratios (PR) by select characteristics in a random sample of adults, Arkansas, May – December 2020.

Characteristics	Past Infections	N	% Prevalence (95% CI)	Crude PR (95% CI)	Multivariable PR (95% CI)
All participants	107	1,565	7.1 (5.8, 8.7)	--	--
**TIME**					
Prevalence during					
May-August	13	422	3.3 (1.9, 5.9)	1 (referent)	1 (referent)
September-October	50	801	6.2 (4.6, 8.4)	1.9 (1.0, 3.6)	1.8 (1.0, 3.4)
November-December	44	342	14.2 (10.4, 19.0)	4.2 (2.2, 8.1)	3.6 (1.9, 6.9)
Total	107	1,565		*P < 0.0001	*P = 0.0001
**PERSON**					
Age (yrs.)					
18-49	69	817	9.3 (7.2, 12.0)	2.0 (1.3, 3.0)	1.7 (1.1, 2.6)
50+	38	748	4.7 (3.4, 6.4)	1 (referent)	1 (referent)
Total	107	1,565		P = 0.001	*P = 0.02
Sex					
Women	74	989	8.2 (6.5, 10.4)	1.4 (0.9, 2.1)	-
Men	33	576	6.0 (4.2, 8.5)	1 (referent)	-
Total	107	1,565		P = 0.13	
Race/Ethnicity					
Non-Hispanic Whites	71	1,255	5.7 (4.4, 7.3)	1 (referent)	1 (referent)
Non-Hispanic Blacks	18	195	9.3 (5.7, 14.6)	1.6 (1.0, 2.8)	1.4 (0.8, 2.4)
Hispanics	15	91	17.6 (10.3, 28.3)	3.1 (1.7, 5.4)	2.3 (1.3, 4.0)
Other	3	24	11.5 (3.4, 32.3)	2.0 (0.6, 6.6)	2.3 (0.8, 6.8)

Total	107	1,565		P < 0.005	P < 0.05
Education					
Without College	75	873	8.7 (6.9, 11.1)	1.8 (1.1, 2.7)	1.6 (1.0, 2.4)
College+	32	689	5.0 (3.4, 7.2)	1 (referent)	1
Total	107	1,562†		P < 0.01	P < 0.05
Occupation					
High-demand essential services**	18	108	19.3 (11.9, 29.7)	2.8 (1.6, 4.9)	2.5 (1.5, 4.1)
Other workers	36	619	5.7 (4.0, 8.1)	0.8 (0.5, 1.3)	0.7 (0.4, 1.1)
Not working***	53	838	6.8 (5.1, 9.1)	1 (referent)	1 (referent)
Total	107	1,565		P = 0.02	P = 0.0001
Any chronic disease					
Yes	57	813	6.4 (4.9, 8.3)	0.8 (0.5, 1.2)	-
No	50	752	7.9 (5.9, 10.6)	1 (referent)	-
Total	107	1,565		P = 0.3	
BMI category					
Underweight (<18.5)	2	23	7.3 (1.7, 26.6)	1.3 (0.3, 5.7)	-
Normal (18.5-24)	16	325	5.8 (3.3, 8.3)	1 (referent)	-
Overweight (25-29)	27	426	7.0 (4.7, 10.5)	1.2 (0.6, 2.4)	-
Obese I (30-34)	27	347	8.1 (5.5, 11.9)	1.4 (0.7, 2.7)	-
Obese II (35-39)	16	219	7.0 (4.1, 11.8)	1.2 (0.6, 2.6)	-
Obese III (40+)	19	225	8.0 (5.0, 12.6)	1.4 (0.7, 2.8)	-
Total	107	1,565		P = 0.9	
**PLACE**					
Urban/Rural Residence					
Rural	44	509	8.6 (6.3, 11.6)	1.4 (0.9, 2.1)	-
Urban	57	992	6.2 (4.7, 8.2)	1 (referent)	-
Total	101	1,501†		P = 0.2	
Region					
Northwest	29	461	7.1 (4.8, 10.3)	1.2 (0.7, 2.1)	-
Northeast	24	249	7.8 (5.2, 11.7)	1.4 (0.8, 2.4)	-

Central	28	580	5.7 (3.8, 8.4)	1 (referent)	-
Southwest	13	96	13.6 (7.6, 23.3)	2.4 (1.2, 4.7)	-
Southeast	7	115	6.1 (2.8, 13.1)	1.1 (0.4, 2,6)	-
Total	101	1,501†		P = 0.2	
Income of Zip Code of residence (USD in thousands)					
1st. tertile (18-36)	49	535	8.9 (6.6, 12.0)	1.8 (1.1, 3.0)	-
2nd. tertile (37-46)	27	468	6.8 (4.5, 10.1)	1.4 (0.8, 2.5)	-
3rd. tertile (47+)	25	498	4.4 (2.6, 6.2)	1 (referent)	-
Total	101	1,501†		*P = 0.02	
Contact with someone known to have SARS-CoV-2 infection					
Yes within household	12	41	29.3 (16.6, 46.3)	6.6 (3.1, 14.1)	4.7 (2.1, 10.1)
Yes, outside household	16	128	13.1 (7.8, 21.3)	2.9 (1.4, 6.2)	2.6 (1.2, 5.7)
No, household size 1+	66	1,128	6.3 (4.9, 8.2)	1.4 (0.8, 2.6)	1.4 (0.7, 2.6)
No and living alone	13	263	4.4 (2.5, 7.7)	1 (referent)	1 (referent)
Total	101	1,561†		*P < 0.001	*P < 0.0005

All P-values of categorical variables are derived from Chi square F Wald Tests except when noted (*) where the P-value is from a Cochran-Mantel F Wald test for trend treating the variable as ordinal.

** Medical assistants, Childcare workers, Personal care aids, Nursing assistants, Police and Sheriff's Patrol Officers, Registered Nurses, Lifeguards, Ski Patrol, and Other Recreational Protective Service Workers.

***Unpaid work including Homemakers, Retirees, Insufficient information.

†3, 64, and 5 records missing data on education, zip code of residence and household size, respectively. These records were retained as a separate category not shown.

-- Variable not included in the final model

Fig 1. Prevalence of SARS-CoV-2 Infection in adults by date of collection, Arkansas, May - December 2020

After adjusting the May to December period prevalence for imperfect sensitivity, the estimate increased slightly from 7.1% to 7.6% (95% CI: 6.2%-9.3%). The corresponding misclassification-adjusted prevalence for November-December increased from 14.2% to 15.1% (95% CI: 11.1%, 20.2%). The adjusted prevalence represents 348,000 adults in Arkansas ever infected.

Risk factors for SARS-CoV-2 past infection

Unadjusted results showed the 8-month prevalence of COVID-19 infection was higher among the young, minorities, particularly Hispanics, lower education, low income, high-risk occupation, South-West region of the state, and self-reported contact with an infected person in the same household (Table 1). There were no differences by gender, body mass index, or self-reported chronic disease. Also, an unadjusted comparison found an association with living in a larger household.

The multivariable analyses showed having contact with an infected person in the same household increased the prevalence of infection by almost 5-fold (PR = 4.7; 95% CI: 2.1, 10.1), over twice the prevalence by contact with an infected person outside the household (PR = 2.6; 95% CI: 1.2, 5.7). Increased prevalence was also found for November-December (PR = 3.6; 95% CI: 1.9, 6.9) and fall months for data collection (PR = 1.8; 95% CI: 1.0, 3.4) compared to the summer. Increased prevalence was also found for work in an essential occupation (PR: 2.5; 95% CI:1.5, 4.1), less than a college education (PR = 1.6; 95% CI: 1.0, 2.4), younger age (PR = 1.7; 95% CI: 1.1, 2.6) and race/ethnicity (PRs 1.4 and 2.3 for NH-Blacks, and Hispanics, respectively). The Hosmer Lemeshow F-goodness of fit test indicated the model fit the data well (*P*-value = 0.2).

Discussion

The study used data from a state-wide probability sample with an acceptable response rate and a clear case-definition. In multivariable analyses, we found COVID-19 infection was associated with race/ethnicity, affecting disproportionately Blacks and Hispanics. Additionally, persons with lower education, who worked in an essential occupation, had contact with an infected person inside the household, or had contact with an infected person outside the household were more likely to be seropositive. The analyses also showed a four-fold increase in COVID-19 prevalence from the first two months in which data were collected to November/December. The imperfect sensitivity adjusted estimate of infection by early December indicates 348,000 infections in adults, or 183,000 more than identified through testing in the state. The difference is considerably lower than results reported by Angulo *et al*. (34), based on earlier US surveys. The difference between surveys in other states and ours may reflect the increased testing capacity in Arkansas during the second half of 2020. Our prevalence point estimate is considerably higher than estimates achieved using a survey of residual bloods from healthcare clinics in Arkansas (9.2%, 95 % CI = 7.2%, 11.1%) (35).

Our study found race/ethnicity was associated with higher COVID-19 infection. Higher infections among Hispanics and Blacks have been documented in several cross-sectional US studies (5-8,18). The prevalence of SARS-CoV-2 infection for Arkansans working in an occupation categorized as high-risk was three-times the prevalence of infection for Arkansans working in other occupations. However, essential

workers in Arkansas had almost three-times the prevalence of infection compared to those not working.

The relation between race/ethnicity, occupation, and socioeconomic status requires further exploration. The distribution of essential workers by race/ethnicity may explain to some degree the observed racial and ethnic associations (36).

Our findings highlight the significant role of household contacts, as well as non-household contacts, in COVID-19 infection. Our results suggest that greater focus should be placed on household spread. This finding is particularly troubling as children and young adults have returned to school. The nature of the transmission can be better characterized using cohort studies of households (37, 38). A study conducted in Guangzhou, the most populated city in southern China, found larger secondary attack rates among household contacts of a primary infectious case (16%-24%), than among non-household contacts (7%-9%) (38). Our findings are also consistent with those studies conducted using a national US sample (10), and a sample collected in New York City (18).

This study is subject to several limitations including the number of and potential misclassification of exposures. Although infections represent only new occurrences since the start of the pandemic, the cross-sectional design could be affected by temporal ambiguity when assessing the role of some risk factors, such as occupation. Because of limited recall of contact with persons with COVID-19 infections, or lack of information on the number of bedrooms per household to appropriately assess crowding, or assigning income based on zip code of residence, there might be some unknown degree of measurement error for some exposure variables. The study

findings may not be generalizable to the entire population as it did not include children nor high-risk institutionalized populations (e.g., prisons, nursing homes).

In summary, the level of humoral natural immunity acquired through infection in a US, mostly rural, southern state by December 2020, before the COVID-19 vaccination started, was 15.1%, In addition, by July 4, 2021, 1,064,000 adults (39) or only 46% of the population of the State, was fully vaccinated. The large proportion of susceptible population, and a more transmissible strain -the Delta variant (B.1.617.2) (40), accounting for most infections in Arkansas in the summer of 2021- may explain this fourth wave experienced at that time.

Acknowledgments

Other members of the Arkansas Coronavirus Antibodies Seroprevalence Survey were Marianne Kouassi, BS, Ryan Mann, BS, Atul Kothari, MD, Hoda Hagrass, MD, PhD. The work was supported through a research contract agreement with the Arkansas Department of Health with funding from the 2020 Coronavirus Relief Fund - CARES Act and by grant UL1 TR003107 from the National Center for Advancing Translational Sciences (NCATS).

Conflict of interest

None.

Data availability statements

The data that support the findings will be provided upon request.

References

1) Angulo FJ, Finelli L, Swerdlow DL. Reopening Society and the Need for Real-Time Assessment of COVID-19 at the Community Level. JAMA. 2020; 323: 2247–2248. doi:10.1001/jama.2020.7872

2) Appa A, Takahashi S, Rodriguez-Barraquer I, Chamie G, Sawyer A, Consortium C, et al. Universal PCR and antibody testing demonstrate little to no transmission of SARS-CoV-2 in a rural community. medRxiv [Preprint]. 2020:2020.08.15.20175786. doi:10.1101/2020.08.15.20175786

3) Feehan AK, Velasco C, Fort D, Burton JH, Price-Haywood EG, Katzmarzyk PT, Garcia-Diaz J, et al. Racial and Workplace Disparities in Seroprevalence of SARS-CoV-2, Baton Rouge, Louisiana, USA. Emerg Infect Dis. 2021; 27: 314–7. doi:10.3201/eid2701.203808

4) Feehan AK, Fort D, Garcia-Diaz J, Price-Haywood EG, Velasco C, Sapp E, et al. Seroprevalence of SARS-CoV-2 and Infection Fatality Ratio, Orleans and Jefferson Parishes, Louisiana, USA, May 2020. Emerg Infect Dis. 2020; 26: 2766-2769. doi:10.3201/eid2611.203029

5) Biggs HM, Harris JB, Breakwell L, Dahlgren FS, Abedi GR, Szablewski CM, et al. Estimated Community Seroprevalence of SARS-CoV-2 Antibodies - Two Georgia Counties, April 28-May 3, 2020. Morb Mortal Wkly Rep. 2020; 69: 965-970. doi:10.15585/mmwr.mm6929e2

6) Menachemi N, Yiannoutsos CT, Dixon BE, Duszynski TJ, Fadel WF, Wools-Kaloustian KK, et al. Population Point Prevalence of SARS-CoV-2 Infection

Based on a Statewide Random Sample - Indiana, April 25-29, 2020. Morb Mortal Wkly Rep. 2020;69 :960-964. doi:10.15585/mmwr.mm6929e1.

7) Chan PA, King E, Xu Y, Goedel W, Lasher L, Vargas M, et al. Seroprevalence of SARS-CoV-2 Antibodies in Rhode Island From a Statewide Random Sample. Am J Public Health. 2021;111: 700-703. doi:10.2105/AJPH.2020.306115

8) Bruckner TA, Parker DM, Bartell SM, Vieira VM, Khan S, Noymer A, et al. Estimated seroprevalence of SARS-CoV-2 antibodies among adults in Orange County, California. Sci Rep. 2021;11: 3081. doi:10.1038/s41598-021-82662-x

9) Demonbreun AR, McDade TW, Pesce L, Vaught LA, Reiser NL, Bogdanovic E, et al. Patterns and persistence of SARS-CoV-2 IgG antibodies in Chicago to monitor COVID-19 exposure. JCI Insight. 2021:146148. doi:10.1172/jci.insight.146148

10) Nash D, Rane M, Chang M, Kulkarni SG, Zimba R, You W, et al. Recent SARS-CoV-2 seroconversion in a national, community-based prospective cohort of U.S. adults. medRxiv [Preprint]. 2021 Feb 16:2021.02.12.21251659. doi:10.1101/2021.02.12.21251659

11) Stout RL, Rigatti SJ. Seroprevalence of SARS-CoV-2 Antibodies in the US Adult Asymptomatic Population as of September 30, 2020. JAMA Netw Open. 2021 Mar 1; 4: e211552. doi:10.1001/jamanetworkopen.2021.1552

12) Santarelli A, Lalitsasivimol D, Bartholomew N, Reid S, Reid J, Lyon C, et al. The seroprevalence of SARS-CoV-2 in a rural southwest community. J Osteopath Med. 2021;121: 199-210. doi:10.1515/jom-2020-0287

13) McLaughlin CC, Doll MK, Morrison KT, McLaughlin WL, O'Connor T, Sholukh AM, et al. High Community SARS-CoV-2 Antibody Seroprevalence in a Ski Resort Community, Blaine County, Idaho, US. Preliminary Results. medRxiv [Preprint]. 2020 Jul 21:2020.07.19.20157198. doi:10.1101/2020.07.19.20157198

14) Bendavid E, Mulaney B, Sood N, Shah S, Bromley-Dulfano R, Lai C, Weissberg Z, et al. COVID-19 antibody seroprevalence in Santa Clara County, California. Int J Epidemiol. 2021 Feb 22:dyab010. doi:10.1093/ije/dyab010

15) Sood N, Simon P, Ebner P, Eichner D, Reynolds J, Bendavid E**,** et al. Seroprevalence of SARS-CoV-2-Specific Antibodies Among Adults in Los Angeles County, California, on April 10-11, 2020. JAMA*.* 2020; 323: 2425-2427. doi:10.1001/jama.2020.8279

16) Bryan A, Pepper G, Wener MH, Fink SL, Morishima C, Chaudhary A, et al. Performance Characteristics of the Abbott Architect SARS-CoV-2 IgG Assay and Seroprevalence in Boise, Idaho. J Clin Microbiol. 2020; 58:e00941-20. doi:10.1128/JCM.00941-20

17) Rosenberg ES, Tesoriero JM, Rosenthal EM, Chung R, Barranco MA, Styer LM, et al. Cumulative incidence and diagnosis of SARS-CoV-2 infection in New York. Ann Epidemiol. 2020; 48:23-29.e4. doi:10.1016/j.annepidem.2020.06.004.

18) Pathela P, Crawley A, Weiss D, Maldin B, Cornell J, Purdin J, et al. Seroprevalence of SARS-CoV-2 following the largest initial epidemic wave in the United States: Findings from New York City, May 13-July 21, 2020. J Infect Dis. 2021:jiab200. doi:10.1093/infdis/jiab200

19) Levy P, Lemeshow S. Sampling of Populations: Methods and Applications, 4th ed., New York: Wiley; 2013 pp.18.

20) Rothman KJ, Greenland S, Lash TL editors**.** Modern Epidemiology. 3rd ed. Philadelphia, PA: Lippincott Williams & Wilkins, 2008. pp. 97.

21) Lin YC, Lee YL, Cheng CY, Tseng WP, Wu JL, Lin CH, et al. Multicenter evaluation of four immunoassays for the performance of early diagnosis of COVID-19 and assessment of antibody responses of patients with pneumonia in Taiwan. J Microbiol Immunol Infect. 2021: S1684-1182 00046-3. doi:10.1016/j.jmii.2021.02.003

22) Schuirmann DJ. A comparison of the two one-sided tests procedure and the power approach for assessing the equivalence of average bioavailability*. J* Pharmacokinet Biopharm*.* 1987; 15: 657-80. doi:10.1007/BF01068419

23) Pasek J. Anesrake: ANES Implementation. R package version 0.80. April 28, 2018. https://CRAN.R-project.org/package=anesrake

24) U.S. Census Bureau, Population Division. Age, Sex, Race, and Hispanic Origin - 6 race groups (5 race alone groups and one multiple race group) (SC-EST2019-ALLDATA6). 2021.https://www2.census.gov/programs-surveys/popest/tables/2010-2019/state/asrh/sc-est2019-alldata6.csv Accessed April 15, 2021

25) Brogan DJ. Software for Sample Survey Data, Misuse of Standard Packages. In: Armitage P, Colton T. eds. *Encyclopedia of Biostatistics*. 2nd ed. Hoboken, NJ: Wiley InterScience,2005. doi.org/10.1002/0470011815.b2a16072.

26) Kleinbaum DG, Kupper LL, Morgenstern H. Epidemiologic Research. Principles and Quantitative Methods. New York: Van Nostrans Reinhold. 1982. pp. 120.

27) Sempos CT, Tian L. Adjusting Coronavirus Prevalence Estimates for Laboratory Test Kit Error. *Am J Epidemiol.* 2021; 190: 109-115. doi:10.1093/aje/kwaa174

28) U.S. Office of Management and Budget, Bulletin No. 15-01, Revised Delineations of Metropolitan Statistical Areas, Micropolitan Statistical Areas, and Combined Statistical Areas, and Guidance on Uses of the Delineations of These Areas,https://www.whitehouse.gov/sites/whitehouse.gov/files/omb/bulletins/2015/15-01.pdf Accessed April 14, 2021.

29) U.S. Centres for Disease Control and Prevention’s National Institute of Safety and Health**.** NIOSH Industry and Occupation Computerized Coding System. https://csams.cdc.gov/nioccs/ Accessed on April 14, 2021

30) Bieler GS, Brown GG, Williams RL, Brogan DJ. Estimating model-adjusted risks, risk differences, and risk ratios from complex survey data. Am J Epidemiol. 2010; 171: 618-23. doi:10.1093/aje/kwp440

31) Mantel, N., Haenszel W. Statistical Aspects of the Analysis of Data from Retrospective Studies of Disease JNCI 1959: 22: 719–48. http://www.ncbi.nlm.nih.gov/pubmed/13655060

32) Shah BV, Barnwell BG. Hosmer-Lemeshow goodness of fit test for survey data. In Proceedings of the Joint Statistical Meetings - Section on Survey Research Methods. San Francisco, 2003.

http://www.asasrms.org/Proceedings/y2003/Files/JSM2003-000744.pdf Accessed April 15, 2020.

33) Vandenbroucke JP, von Elm E, Altman DG, Gøtzsche PC, Mulrow CD, Pocock SJ, et al. Strengthening the Reporting of Observational Studies in Epidemiology (STROBE): Explanation and Elaboration. PLoS Med 1007; 4: e297. doi.org/10.1371/journal.pmed.0040297

34) Angulo FJ, Finelli L, Swerdlow DL. Estimation of US SARS-CoV-2 Infections, Symptomatic Infections, Hospitalizations, and Deaths Using Seroprevalence Surveys. JAMA Netw Open. 2021; 4: e2033706. doi:10.1001/jamanetworkopen.2020.33706

35) U.S. Centres for Disease Control and Prevention. Nationwide Commercial Laboratory Seroprevalence Survey. Accessed August 31, 2021. https://covid.cdc.gov/covid-data-tracker/#national-lab

36) Webb Hooper M, Nápoles AM, Pérez-Stable EJ. COVID-19 and Racial/Ethnic Disparities. JAMA. 2020; 323: 2466-2467. doi:10.1001/jama.2020.8598

37)He Z, Ren L, Yang J, Guo L, Feng L, Ma C, et al. Seroprevalence and humoral immune durability of anti-SARS-CoV-2 antibodies in Wuhan, China: a longitudinal, population-level, cross-sectional study. *Lancet.* 2021;397(10279):1075-1084. doi:10.1016/S0140-6736(21)00238-5

38) Jing QL, Liu MJ, Zhang ZB, Fang LQ, Yuan J, Zhang AR, et al. Household secondary attack rate of COVID-19 and associated determinants in Guangzhou, China: a retrospective cohort study. Lancet Infect Dis. 2020:1141-1150. doi:10.1016/S1473-3099(20)30471-0.

39) United States Centers for Disease Control and Prevention. COVID-19 Data Tracker, Trends in Number of COVID-19 Vaccinations in the US. https://covid.cdc.gov/covid-data-tracker/#vaccination-trends_vacctrends-fully-cum Accessed August 31, 2021.

40) Allen H, Vusirikala A, Flannagan J, Twohig KA Zaidi A Groves N, et al. Increased household transmission of COVID-19 cases associated with SARS-CoV-2 Variant of Concern B.1.617.2: a national case-control study. Public Health England. 2020. https://khub.net/documents/135939561/405676950/Increased+Household+Transmission+of+COVID-19+Cases+-+national+case+study.pdf/7f7764fb-ecb0-da31-77b3-b1a8ef7be9aa Accessed September 14, 2021.

Fig 1

Click here to access/download;Figure;Fig1.tif

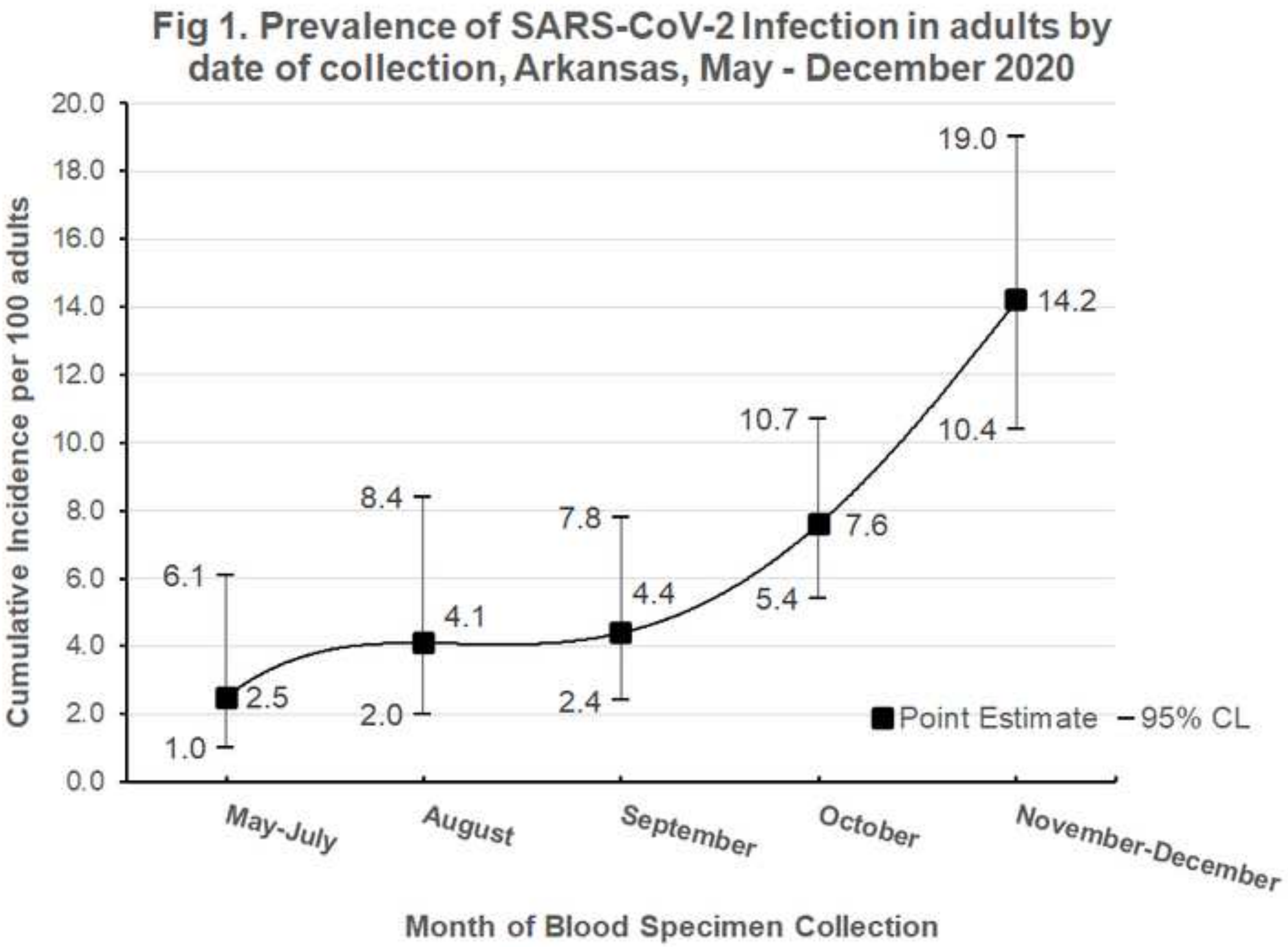

Contribution of Authors

Click here to access/download

Supporting Information

Authorship statement.docx

Click here to access/download
Supporting Information
STROBE_checklist_v4_combined.docx
