# Supplementary material for: State-wide random seroprevalence survey of SARS-CoV-2 past infection in a southern US State, 2020
Source: PLoS One. 2022 Apr 27;17(4):e0267322. doi: 10.1371/journal.pone.0267322 (PMC9045671; doi:10.1371/journal.pone.0267322)
Supplement: Attachment — Submitted filename: Authorship statement (2).docx [file pone.0267322.s007.docx]

**Authorship statement**

Conceptualization- Drs. Amick, Williams, Cardenas, Fischbach, James, Kennedy and Gardner contributed to the conceptualization of the Arkansas CASS project.

Data curation – Ms. Boothe and Dr. James supervised the interview data acquisition. Dr. Kennedy prepared data from his laboratory and compared them with those of Dr. Olgaard, who ran the laboratory experiments, conducting the Beckman Coulter high throughput chemiluminescence assays. Dr. Du curated and merged the questionnaire and laboratory data and obtained the raking weights. Dr. Cardenas acquired the occupation and industry coding, and rural/urban from raw interview data and prepared the analytic files.

Formal Analysis – Dr. Cardenas carried the analysis with input from Drs. Amick, Nembhard, Du and Zoohori.

Funding acquisition – Drs. Gardner, Williams, Amick, James Kennedy, and Zoohori contributed to the acquisition of funding.

Investigation – All authors kept up to date bibliographic research on this topic, Dr. Cardenas shared a bibliographic database with other team members. Drs. Cardenas and James obtained and maintained IRB approval and documentation.

Methodology – Drs. Cardenas and Fischbach developed the protocol and manual of standard operational procedures; consulting with Drs. Kennedy and Olgaard with the laboratory methods. Drs. Fischbach, Cardenas and Amick developed the questionnaire. Ms. Boothe contributed to field testing the questionnaire. Dr. Cardenas developed and implemented plan of analyses, with input from Drs. Du, Nembhard, Amick, Zohoori and Ms. Jin.

Project administration -Drs. Gardner, Williams, and James maintained weekly meetings of the study team which steered the study over the 18 month-period. Drs. Amick and Williams identified and negotiated with different vendors such as Dynata the terms of references.

Resources – Dr. James provided support of the UAMS Translational Research Institute to support the development of the RedCap application used in data collection and data management. Drs. Kennedy, Boehm, Forrester and Olgaard provided human resources to carry out the laboratory procedures cited in the text, in addition to other measurements related to the project.

Software – REDCap, SAS, SUDAAN and R as cited in the text.

Supervision - Dr. Amick supervised the team carrying out the screening calls. Ms, Boothe contributed to this effort for several months. Drs Kennedy and Olgaard supervised the laboratory work, while Drs. James, Fischbach, Cardenas, Ms. Booth supervised field collection.

Validation – Drs. Kennedy, Boehme and Forrester assessed the reproducibility of the results of the Beckman Coulter high throughput chemiluminescence assays against in-house immunoassays.

Visualization – Dr. Cardenas prepared figure 1 in the manuscript, edited by Dr. Zoohori.

Writing –Dr. Cardenas prepared the original draft.

Writing – review & editing. Drs. Amick, Williams, Nembhardt, Zoohori extensively edited several versions of the draft, and final edits were provided by Drs. Olgaard, Du, Jin, James and Kennedy. All authors read and approved the final version of this manuscript.
