# Supplementary material for: State-wide random seroprevalence survey of SARS-CoV-2 past infection in a southern US State, 2020
Source: PLoS One. 2022 Apr 27;17(4):e0267322. doi: 10.1371/journal.pone.0267322 (PMC9045671; doi:10.1371/journal.pone.0267322)
Supplement: Attachment — Submitted filename: Point-by-pointResponsePLOSOneFebruary10.doc [file pone.0267322.s008.doc]

**Re: PONE-D-21-30543**

**Title:	State-wide random seroprevalence survey of SARS-CoV-2 past infection in a southern US State, 2020**

**Responses to Reviewer’s Comments and Suggestions**

**Reviewer 1**

We would like to thank the reviewer for the valuable input and suggestions.

**First point**

*This study focused on estimating the number of Arkansans residents infected with SARS-CoV-2 between May and December 2020 and also to assess the determinants of infection. This was carried out by surveying the seroprevalence of, a statewide population-based random-digit dial sample of non-institutionalized adults in Arkansas. The outcome was past Covid-19 infection measured by serum antibody test . Notably, the seropositivity was significantly elevated among non-Hispanic black , (and) Hispanic.*

*The method on how All sera were tested for IgG antibodies that target receptor binding domain of the spike protein of the SARS CoV-2 using the Beckman Coulter DxI instrument should be explained.*

**Response-** We are very appreciative of the summary of this reviewer as it reflects the nature and importance of our study. We have tried to explain better the method used to test for the IgG antibodies to the RBD of the spike protein of the SARS-CoV-2.

We have added the following to the revised version:

“The outcome variable was evidence of COVID-19 infection as measured by a positive clinical laboratory test. All sera were tested for IgG antibodies that target receptor binding domain of the spike protein 1 (S1) of the SARS CoV-2 using the Beckman Coulter DxI instrument (Brea, CA; Access SARS-CoV-2 IgG chemiluminescence immunoassay) in a CLIA certified clinical laboratory. In this automated instrument’s two-step immunoassay, the subjects’ serum samples were added to a mixture of buffer and paramagnetic particles coated with a recombinant SARS-CoV-2 spike protein specific to the S1 receptor binding domain. Following incubation, unbound protein is washed away, and anti-human IgG alkaline phosphatase conjugate monoclonal antibody is added. A second wash removes unbound conjugate. A chemilumiscent substrate is then added and the amount of light emitted is read using a luminometer…”

**Major Criticisms**

*Overall, the data is promising, but the novelty of this study is relatively weak because the outcomes of the results obtained is not clearly explained.*

**Response-** As suggested, we have emphasized that non-random samples, for example, convenience samples are more likely to be affected by selection bias. By comparing our results with those of a survey of residual bloods from healthcare clinics in Arkansas we found significant differences: 9% of past infection in residual samples obtained in December 2020 versus 14% in our survey.

The revised text reads:

Introduction: “We aimed to assess the proportion of the population susceptible to SARS-CoV-2 infection in a representative sample of the adult population in Arkansas in 2020, as opposed to those derived from convenience samples more likely affected by selection bias.”

Discussion: “Our finding provides some support to the notion that convenience samples are more likely to be influenced by selection bias than population-based samples.”

“This study informed the public and state health authorities that the population of Arkansas remained mostly susceptible (i.e., 85%, or 100% – 15%) to SARS-CoV-2 infection by the end of 2020. The introduction of more transmissible strains such as the Delta variant (B.1.617.2) (40) by the summer of 2021 with vaccination primarily targeting high-risk groups largely explains the fourth wave experienced at the time of the submission of this manuscript.”

**Minor issues:**

*1. The introduction section needs to be worked on and be improved for example*

*Of these, only seven used random sampling procedures so that every person in the target population had “a known, non-zero probability of being included in the sample*

*A comma interferes with the flow.*

**Response-** The text is quoted from the textbook of Paul Levy and Stan Lemeshow, and the comma separates two items. The first is that the probability is known, and second the is not zero:

**“**a known, non-zero probability..” We thank you for the observation.

*"* *The data obtained in this work are of interest for infectious disease specialists. The research was carried out using adequate methods and the manuscript may be published.*

**Response-** Thanks for your comment.:

**Reviewer 2**

*“This was an important study and authors conducted it well.”.*

**Response-** We are thankful for comment.

*Agreeing with the study's limitation of not including children. The study should have included children as they also play a crucial role in the transmission of SARS-CoV-2 infections; and it would have been nice to also learn if factors oberved in adults were also similar to those of children.*

**Response-** We agree with the reviewer.

*Authors should pay more attention to their references, consistency should be applied.*

**Response-** We have checked for consistency and used the PLoS One guidelines.

*Manuscript should be checked for editorials and should also be checked by an English expect.*

**Response-** We have checked for potential spelling and grammar errors.
